# Homeowner perspectives on the implementation of the Community Homes for Opportunity (CHO) program: an ethnographic group homes study in Southwestern Ontario Canada

**DOI:** 10.1186/s12889-023-15512-2

**Published:** 2023-03-29

**Authors:** Cheryl Forchuk, Sebastian Gyamfi, Heba Hassan, Bryanna Lucyk, Richard Booth

**Affiliations:** 1 Beryl and Richard Ivey Research Chair in Aging, Mental Health, Rehabilitation and Recovery, Lawson Health Research Institute, Arthur Labatt School of Nursing, Western University London, Parkwood Institute Mental Health Care Building, 550 Wellington Road, Suite B3-110, P.O. Box 5777, London, STN B, N6A 4V2 Canada; 2 Lawson Health Research Institute, Arthur Labatt School of Nursing, Parkwood Research Institute, Western University, London, Canada; 3 Lawson Health Research Institute, Parkwood Research Institute, London, ON Canada; 4 Lawson Health Research Institute, Arthur Labatt School of Nursing, Western University, London, Canada

**Keywords:** CHO, HSC, Homeowners, Housing, Autonomy, Recovery, Community integration, Supportive housing

## Abstract

**Background:**

The global extant literature acknowledge that housing serves as a key social determinant of health. Housing interventions that involve group homes have been found to support the recovery of persons with mental illness and those with addiction issues. The current study explored the views of homeowners in relation to a supportive housing program called Community Homes for Opportunity (CHO) that modernised a provincial group home program (Homes for Special Care [HSC]) and provided recommendations for improving the program implementation in other geographical areas of Ontario.

**Methods:**

We applied ethnographic qualitative techniques to purposefully recruit 36 homeowner participants from 28 group homes in Southwest Ontario, Ontario Canada. Focus group discussions were conducted at two time points, during CHO program implementation (Fall 2018, and post implementation phases (Winter 2019) respectively.

**Results:**

Data analysis yielded 5 major themes. These include: (1) general impressions about the modernization process, (2) perceived social, economic and health outcomes, (3) enablers of the modernization program, (4) challenges to implementation of the modernization program, and (5) suggestions for implementation of the CHO in future.

**Conclusions:**

A more effective and expanded CHO program will need the effective collaboration of all stakeholders including homeowners for successful implementation.

## Background

The global extant literature acknowledge that housing serves as a critical social determinant of health [1–5]. Yet, people with significant mental illness and addiction problems sometimes have trouble attaining and maintaining housing. Poor housing conditions negatively affect tenants' health [6–9] and well-being [2]. Recovery-oriented programs such as supportive housing interventions that provide coordinated assistance for persons with mental illness, including addiction, result in significant improvements in health-related outcomes and recovery [6, 10–12] and effective community integration [13, 14].

Supportive housing is a program where staff provide various support services within the tenants’ residences. Supportive housing is provided in multiple housing types, including group homes, clustered apartments, and halfway homes [15, 16]. Overall, supportive housing is cost-effective [17], reduces frequent (re)hospitalizations [18], enhances independence, and produces positive psychosocioeconomic outcomes to help improve the quality of life of persons with serious mental illness and addiction [19].

Supportive housing models are guided by housing first principles to aid recovery with less emphasis on pathology while emphasizing rehabilitation. Supportive housing frameworks also foster individual strength-building where the consumer is seen as an expert and part of the shared decision-making process that promotes autonomy in the end [20]. Overall, decisions are based on the individual’s experiences and needs [21, 22] and the provider’s expertise in working together [22]. As indicated in the housing first philosophy, five core principles guide the planning and implementation of supportive housing programs. These principles include (i) immediate access to permanent housing without any housing readiness requirements, (ii) as a right, the individual makes their own choice of housing, (iii) recovery-oriented, (iv) recognizing the individuality of clients concerning supports, and (v) assist clients in engaging in social activities for community integration.

The current study (Community Homes for Opportunity [CHO]) falls within the confines of supportive housing and applies Housing First principles to provide housing stability and enhanced community integration for tenants. The overall goal of the CHO is to assist tenants in integrating into the community. The CHO is a person-centered community-based housing model that offers institutional care through meals and 24-h staff support. The CHO program promotes tenants’ recovery, autonomy, and community integration while encouraging them to participate actively in their care regarding goal setting, planning, and individual choices.

Supportive housing programs such as the CHO that provide combined formal (off-site healthcare providers) and informal on-site supports that include homeowners, and their staff, are effective in reducing homelessness [21, 23–25]. Brackertz et al. [10] report that supportive housing programs yield more positive outcomes if coordination at the state or local levels, cross-sector collaboration, and partnerships among stakeholders and Landlords are effective. According to Brackertz and colleagues, developing healthy relationships such as engaging, educating, and frequent communication with landlords will build their capacity and help mitigate some inherent challenges, including financial difficulties, staff problems, and the nonavailability of beds associated with housing available for tenants. Accordingly, for optimum outcomes, strong relationships should exist among landlords and the network of tenants (consumers), clinicians (staff), and managers. Such effective relationships have been found to encourage landlords to readily consult with the rest of the service team members when problems arise in the homes [10].

The public health impact of unstable housing on persons with mental illness, including addiction problems, is well documented in the extant literature [2, 6–9]. What we do not know is the perspectives of the homeowners (landlords) who play a significant role in ensuring that appropriate housing with adequate conditions is available for these tenants. Therefore, we explored homeowners’ views concerning the CHO and related policies and practices to provide recommendations for improving the program implementation in other geographical areas in Ontario.

## Materials and methods

### Study context

This study was part of a larger mixed-methods research project that was conducted to evaluate the ‘Phase One Homes for Special Care (HSC) modernization program’ within 28 homes operating through St. Joseph’s Health Care London within a geographic area encompassing the former catchment areas of London and St. Thomas provincial psychiatric hospitals in Ontario, Canada. This evaluation examined the nature and experiences of homeowners (Private operators of the homes that adopted the CHO program) in response to the changes being made.

The CHO is a modified version of the Homes for Special Care (HSC) program (a legacy program in Ontario that was established in 1964 by the Ontario Ministry of Health to offer long-term supportive housing in group homes for persons discharged from Provincial Psychiatric Hospitals). The HSC was administered through the hospitals’ former psychiatric system with 24-h availability of staff, meals, supervision, and assistance of tenants with activities of daily living. Although the HSC provided some form of supportive housing, it was more custodial and did not offer tenants autonomy and choice (which are fundamental principles of recovery) [26]. Therefore, the HSC needed modernization that had not occurred in decades. In 2018, the Ministry of Health, St. Joseph’s Health Care, and community partners started the modernization of HSC to become Community Homes for Opportunity (CHO).

Since 2018, the CHO has provided individuals access to subsidized group housing with support where staff with expertise offer care and ongoing assistance to promote growth and independence. To enable tenants to achieve this integration, they receive increased financial support and assistance with life skills through a variety of activities and programs, including furthering education, financial literacy, and personal hygiene, managing their resources, and taking part in leisure activities of their choice within the home and the community. Tenants are also paired with a community social worker for extra support in the home and the community for improved physical and mental health.

### Study design and sample

The research explored the views of homeowners concerning the process and outcomes of the CHO implementation program in South-West Ontario using focused ethnographic techniques. The focused ethnography design was deemed fit for examining the nature and experience of the homeowners because it facilitates a timely investigation of health problems within a relatively short period compared to classical ethnography, which requires engagement over a prolonged period [27–29]. The focused ethnographic method allowed the researchers to involve and engage the homeowners about their experiences and benefits of the CHO program to the tenants and to identify relevant insights concerning policies and practices that foster or interfere with the ongoing housing, which in this case were changes being implemented, under the CHO program.

Twenty-eight homes serving 368 tenants were included in the evaluation of the CHO program implementation. After explaining the purpose of the study and gaining consent, the homeowners whose homes were already part of the CHO implementation program were enrolled in the study. In all, 36 homeowners were enrolled in the study.

### Data collection

The current study was approved by the Western University Health Sciences Research Ethics Board (HSREB). Posters announcing the research were distributed in the CHO homes for participant recruitment. Focus groups were conducted with the homeowners to identify issues, solutions, and recommendations for improving the CHO. We used focus group discussions to gain an in‐depth understanding of the homeowners’ perspectives concerning social change among their tenants and the homes. Before the commencement of data collection, the researchers reiterated the study’s objectives, after which they obtained written informed consent from each participant before starting the focus groups. Focus group discussions took an average of 50 to 90 min each. The focus group discussions dwelled on the following questions: (1) What is the context/circumstance of the home? (2) What are the factors/issues that you perceive as barriers and facilitators while implementing the modernized program? (3) What are the perceived benefits and challenges of implementing the modernized program? (4) What are the modernized program's health, social, and economic outcomes (quality of life, social inclusion, and costs/cost/savings)? (5) What do you recommend to improve the service? Data were audio-recorded and then transcribed verbatim by two research team members. Note-takers gathered information about group dynamics, context, and non-verbal communication, which were later integrated into the transcribed data to augment research findings. The homeowner focus groups were conducted at two-time points. The 36 homeowners who took part in the study participated in 19 discussions, i.e., seven (7) focus groups (*n* = 10) and 12 focus groups (*n* = 26) participants during CHO program implementation (Fall 2018) and post-implementation phases (Winter 2019), respectively. Over the period, focus groups were conducted with different homeowner groups. The participants did not necessarily have to join the same group at the two data collection points. This variety among participants improved the group dynamics while enhancing diverse opinions from the homeowners.

### Data analysis procedure

An ethnographic qualitative analysis developed by Leininger [30] was applied to examine focus group data. We used an inductive process to understand the transcribed focus group data and field notes while attending to what we learned from the data to avoid preconceived ideas concerning the topic under study. The inductive approach also helped the researchers to derive findings in the context of the evaluation questions. Two graduate research assistants analyzed the data. They first listened to the audio tapes to acquaint themselves with the raw data, after which each assistant read through focus group transcripts separately. This helped to identify preliminary codes based on distinct descriptors of ‘what worked well, what did not work well, and suggestions for improvement.’ Each of the researchers applied this technique in analyzing data from each site. Data were first coded with descriptive labels. The identified codes were then categorized alongside their respective exemplar quotes into subthemes based on their meaning concerning the context of participants’ views. The categories were developed into subthemes based on identified meaning.

The researchers analyzed focus group discussions involving CHO program implementation and post-implementation data separately. Having identified all subthemes from each site, the researchers aggregated them based on similarities and differences between the findings about each time point. Ultimately, the emergent themes from the implementation and post-implementation focus group discussions were further aggregated and grouped based on commonalities about facilitators, benefits, challenges, and suggestions for improvement. To ensure the credibility and trustworthiness of the study findings, all members of the study team were given copies of the results to appraise and to make inputs in the form of comments. The primary investigator also organized a public forum to solicit the participants’ views about the findings for comments. In the end, all comments and observations from the participants and co-researchers were integrated into the final research document to form a report.

## Results

The participating homeowners mostly identified as Caucasian (97%). The majority (61%) of these participants were female. About 70% of the homeowner participants were aged 40–59 years. See Table 1 below for details.

**Table 1:** Sociodemographic characteristics of study participants

Demographic Variable	Variable estimates	
	**N**	**Percentage (%)**
**Sex**		
▪ Male	14	38.9
▪ female	22	61
**Age (years)**		
▪ 20–39	7	19.4
▪ 40–59	25	69.4
▪ 60–79	4	11.1
**Ethnicity**		
▪ Caucasian	35	97.2
▪ Other race	1	2.8
**Total N**	**36**	**100**

Data analysis yielded five (5) major themes with several subthemes. The major themes identified included: general impression about the CHO program, perceived social, economic, and health outcomes, enablers of the program implementation, challenges to effective program implementation, and suggestions for the effectiveness of the CHO intervention. See Table 2 below for a summary of the study findings.

**Table 2:** A summary of study findings

Descriptor	Major theme	Subtheme
**What has gone well?**	**General impression about CHO Program**	Positive perceptions about CHO Cordial interpersonal relationships
	**Perceived social, economic and health outcomes**	Improved community involvement and socialization Improved financial support for tenants Noticeable enhancement in quality of life Perceived tenant empowerment
	**Enablers of the CHO intervention**	More collaboration between stakeholders Funds are paid on time
**What has not gone well**	**Challenges to CHO implementation**	Lack of standardized tools Lack of appropriate amenities Financial constraints
		Tenants limited financial literacy Staffing problems
**What do you recommend to improve the service?**	**Suggestions for improving CHO**	Standardizing organizational functioning Increase funding for CHO Transparency and collaboration between stakeholders Addressing power relations and philosophical differences

### General impression about CHO program

The homeowners revealed how they perceived the CHO program. Their views pertained to opinions they had formed during the CHO implementation period in relation to the impact of the program. Some of their impressions include positive perceptions about CHO and cordial interpersonal relationships among tenants, staff, and homeowners.

### Positive perceptions about CHO

Some homeowners expressed positive feelings about how the CHO program was being implemented. They felt they were getting extra coverage under the new housing intervention compared to the old program (the HSC). For instance, under the new housing intervention, homeowners get paid some money when a tenant moves in and when they incur an empty bed due to someone moving out of the house.

This participant expressed;

With the HSC [previous program], it was very centralized. We had restrictive legislation for HSC. With this new program, two things are accomplished. Tenants not only have the backing through the Community Agency, they have the encouragement of the CHO program.

Another participant revealed;

The 60 days payable empty bed is a plus, the transition pay for 90 days is $5/day. When somebody moves in, we get an extra $5/day to compensate for the increase in workload. I feel I have more support to take care of tenants. If [I] have an emergency I know they will support me.

### Working relationships

In addition to the Positive Perceptions about the modernized program, homeowners mentioned the development of healthy interaction between stakeholders such as the CHO workers and the tenants. The CHO staff built good relationships with tenants to the extent that some have been helping tenants in managing their money. Such working relationships and support for tenants contribute immensely to their autonomy vis-à-vis day-to-day life. Healthy relationships between the workers and tenants were vital to homeowners because positive relationships enhance therapeutic relations and tenant experiences of satisfaction, adherence to treatment modalities, and improved quality of life [31, 32]. A participant revealed; “They [referring to CHO workers] have good relationship with them [tenants]. So, regarding CHO I mean … I love our worker (home staff) and the new (Community Agency staff she seems really nice too. I think it’s all smooth”. Another participant added; “[Community Agency staff] help them [tenants] how to use this money, how to order extra money from the public trustee.”

### Perceived social, economic, and health outcomes

#### Improved community involvement and socialization

The modernized program offers the tenants more opportunities to be involved in the community. Even individuals who previously had no interest in taking the bus had started going out on their own more than they did with the previous program (HSC). The enhanced funding enabled the tenants to embark on various social activities that improved their social acceptance and inclusion. This participant revealed; “CHO has rewarded them [Tenants] more opportunity to get into a community and back with transportation. The funding change has a greater effect on this home. So there’s more opportunity”. Another participant said; “There are things that they want to do, and it’s coming out. And even individuals who don’t take the bus are now thinking about taking it”.

#### Improved financial support for tenants

Some homeowners observed that tenants were now getting a lot of financial assistance from the increase in the amount of money. Their tenants had money to do the things they needed to do. For instance, homeowners mentioned that tenants were more receptive to getting engaged in a lot more activities outside the home. Despite this positive observation, some homeowners disagreed with the financial choices of some tenants and commented that tenants were misusing the monies they were receiving (not using the money for the intended purpose). Despite the glitches, the homeowners described the CHO as an incentive for improved engagement in leisure activities for tenants. A participant expressed; “Some clients are enjoying [because of] the increased amount of personal needs allowance. For others, it’s been a wonderful bonus. [They] can manage things well”.

This participant revealed;

We had some problems, the increase in personal needs allowance for the residents has created in a positive way a lot of cases, but it also created some mini monsters around too; people refusing to look after what the money was intended for, example, haircuts. Now they’re saying, ‘I don’t have to’.

#### Noticeable enhancement in quality of life

Despite initial fears among some homeowners about introducing the CHO intervention, focused group discussions with these landlords revealed improvement in the standard of health, comfort, and happiness among tenants. The positive progress with the CHO gave hope to the homeowners about the prospects of the program.

A participant stated;

In terms of modernization, I think it’s critical that it [CHO] continues, not only because of all the work that’s gone into the first portion of it but just from a basic understanding that, from a quality of life perspective there’s been great gains. The positive impacts certainly have outnumbered [the negatives] 2-fold

Another participant said;

We’ve seen a presence [referring to CHO] for a year now and there’s been a huge change in their [tenants] behaviors. Very receptive and opening up. That’s the biggest plus. For sure there’s way more positives than negatives. I’d say, excellent!

#### Perceived tenant empowerment

Homeowners compared the CHO with the HSC. They concluded that the modernized program was more adaptable to the current needs of the tenants in fostering individuality, independence, human rights, and autonomy toward recovery and community integration. Improving tenant autonomy and independence was one of the key underlying principles of the supportive housing project, CHO. According to most homeowners, many tenants began making decisions on their own, and doing things they felt could benefit them. Only a few needed some form of guidance from time to time. Sometimes the landlords needed to consult with the tenants before certain activities were undertaken including food choices, and groceries among others. Such interactions proved healthy for both parties (i.e., homeowners and tenants) in navigating the therapeutic trajectory.

A homeowner said;

So, we’ll say do you have a grocery list that you take? It’s not like ok you’re getting this food, it’s like ‘what would you like, what do you want to eat?’ Yea, like we follow the Canada food guide. So, there’s meat, there’s vegetables and we make sure the meals are balanced

Another participant added;

We’ve provided locked space for each of the tenants here. We did some closet adjustments and put some walls between closets that used to be shared so that everybody can have their own secured locked space. So, we did that, and it’s worked out very well…Yea more independence, more decisions for themselves

### Enablers of the CHO intervention

#### More collaboration between stakeholders

The effective communication processes occurring between stakeholders (homeowners, staff, and the mental health agency) build strong bridges that are good for strengthening service delivery to the clients through easy access and less cumbersome referral procedures to the homes. The community agency acts as a centralized agency, liaising with homeowners and aiding people with mental illness with appropriate support. A homeowner disclosed; “…numbers have been up because there’s more access to more referrals… our homes are filled now with tenants. The fact that there’s cooperation in everybody targeting this new CHO is probably the best thing about it right now”. Another participant intimated; “The Community Agency is taking the referrals in for CHO. So, when they get a referral in, they’re able to now streamline it to the most appropriate housing for that individual. I think it’s beneficial for the clients.”

#### Funds are paid on time

Focused groups with homeowners revealed that some of them were satisfied with the payment mode of their monies even though there was room for improvement. They believed the decentralized system with Community Agencies in the modernized program was more effective in addressing pay issues more quickly than the centralized system at the hospital which previously existed. They were confident prompt payments would go a long way to strengthen their service delivery towards an effective and sustained CHO program. This participant said: “I mean a big challenge for homeowners is pay but that’s been going alright. The funding is fine. I mean they were late a few times in the beginning, but they’ve rectified that”. Another participant added: “We’re not getting paid statements, but money is flowing into our accounts…money is flowing which was a worry in the beginning…we got paid by the ministry as well so this should really be a good thing”.

### Challenges to the CHO implementation

#### Lack of standardized tools

Focus group discussions with homeowners yielded a variety of opinions. For instance, the participants identified the absence of clear guidelines and tools, including job descriptions and responsibilities for workers in the homes, leading to role confusion and, in some instances, strained relationships between some stakeholders. Some participants believed the absence of a uniform structure provincially was hampering the effectiveness of the CHO program.

A participant disclosed;

We argued from the very beginning [that] if you want this to have a provincial feel to it you’ve gotta give provincial structure and you’re gonna build this in collaboration with the agencies. For the basics of how the money is gonna flow and what the forms are gonna look like, and what is and is not okay for an agency to do, there are no framework so we’re seeing such inconsistencies across agencies

Another participant added;

Planning from the beginning was huge and they [Community Agency] made it very clear that CHO would be implemented, and we’d still be building it, you know so, that was made clear to us but even with that statement I think it’s falling short of what everybody should be doing

#### Lack of appropriate amenities

Some homeowners complained that rooms in the home were small and therefore could not fit lockers as was directed by the community agencies. The lack of space in the homes was one of the major challenges affecting tenants’ comfort and privacy. A homeowner disclosed; “You know like I said it depends on the home like my home doesn’t have room for lockers, like you can go and see the rooms how small”.

This homeowner expressed some concerns;

The space in the home is maximized with what we have. We have a kitchen, she [Community Agency staff] can sit in the kitchen with people, she can sit in the dining room with people. That’s generally what she does; she’ll sit at the dining room table and take up the whole table with her papers

#### Financial constraints

Some homeowners who participated in the study complained about the seeming lack of funds and relevant resources for running the home activities. Whiles some bemoaned the lack of incentives, others criticized the funding model due to some inconsistencies in reimbursement. Funding had not increased to match the current cost of living. Homeowners, therefore, had to make an additional budget and pay out of their pocket for repairs and maintenance and additional food due to limited funds.

A homeowner disclosed;

You pull your hair out…we don’t work on volume and we can’t afford it, we can’t afford to pay our staff, and that’s why we can’t keep staff either, it’s a big turnover. The funding is shrinking dramatically with the amount of work we’re having to pick up and do

Another homeowner said;

… You know we have a budget here because we’re very limited with money and that’s another thing too. We’re going to do like a cooking class; to bake cookies or bake whatever and the thing is it’s stuff we don’t use on a regular basis. We don’t have the money for that

#### Tenants limited financial literacy

Focused group discussions revealed the need to offer continuous help to tenants in relation to how they spend their money and their choices. The homeowners also identified a lack of financial literacy. By this, some tenants were found to have difficulty managing their finances. They engaged in wasteful spending (buying things that were largely not contributing positively to health and well-being.

A homeowner expressed this concern;

Before, everything was paid for them[tenants] and we just gave them their spending money which was $150 a month. Now they’re given $500 and they’re having to budget [on] their own? My guys are not so bad, but some of the clients run through their money quicker if they’re heavy smokers and things like that… They’re not buying what they’re supposed to, so that is an issue

Another homeowner disclosed;

It’s been enough months now. We’re seeing a lot of problems with people spending unwisely. If you can go and pick out a brand-new outfit for yourself and purchase it, that gives you pride in your appearance. We’re seeing a lot of smoking way more than before. We’re not seeing the negative health impacts of the extra smoking yet, that’ll be a couple years down the road

#### Staffing problems

Data analysis revealed that most homeowners had difficulty finding suitable staff for their homes. According to the homeowners, financial constraints contributed to the inconsistencies in staffing, especially CHO workers w affecting service delivery and efficiency in the homes. In the end, homeowners feared work overload will make the CHO program look like it is always in transition. This homeowner complained; “That’s a huge issue because now you’re not getting qualified people, you’re not getting good people. I’m lucky to have good people but, especially in small towns like this, trying to find people is impossible”.

Another homeowner echoed:

You’re small homeowners, I guess we’re competing with big agencies that maybe their workers make 22 dollars an hour. We can’t afford to pay and also we can’t retain people because we can’t offer to pay them that money. So a lot of the homeowners live in the homes by themselves

### Suggestions for improving CHO

#### Standardizing organizational functioning

Homeowners expressed the need for structured, across-board policies and activities that include standardization of tools for practice. They also suggested detailed contract agreements from the Ministry, clear policies and guidelines, especially in crisis situations, and consistent inspection rules across all houses including wellness plans. They believed ensuring uniformity will place all homes on the same degree of care and outcome. The lack of clear guidelines and issues with responsibilities, roles, and job descriptions were another concern for most homeowners. They believed setting clear job descriptions, worker rules, and social norms needed to be explicitly stated to the CHO workers from the beginning. The owners were convinced clear role descriptions and guidelines could help prevent conflict between the home staff and the CHO workers.

A homeowner recommended;

I think they should have a list of rules and responsibilities set out. I think that should be the same for everybody. The system should’ve been set up with all the papers, all the policies, procedures in advance, so the ministry basically had to say here’s the Community Agency, here’s everything structured

Another homeowner suggested;

Having defined roles and responsibilities, better communication, and having an outline … it does impact you, impact your clients when you don’t even know your role…Some better understanding for everybody on how things should work in the home and that sort of stuff

#### Increase funding for CHO

The homeowners advocated for more support from the Ministry in the form of an increase in house funds to match the increase in prices, an increase in payment for home staff, and extra funds for homeowners for staffing. They also recommended the need to break down and quantify the amount of funding given to them.

A homeowner suggested;

Our funding formula needs to be looked at in terms of how much funds should be allocated to staffing, maintenance and so on. It should be broken down. Because right [now] they give us a lump sum and then, just say ‘oh you can make it up’. If they break it down they would see that the allowance is not enough. Especially if you go by … like in nursing homes and things like that, they’re allowed $9 a day for food but in jails, it’s 11 or 12 dollars for food. Here I think you do 2 or 3 dollars

#### Transparency and collaboration between stakeholders

Whiles seeking to identify who was solely administering the program homeowners at the same time called for close collaboration and understanding among all stakeholders as well as the need for effective conflict resolution processes. They also urged all interested parties to be more open and approachable to deal with existing power differentials for enhanced teamwork.

This homeowner advised;” For the next roll-out I’d say look at the whole approach. This has been teamwork, and all the service providers, homeowners, the agency, workers, and any agencies in the community should all be looked at as team players”. Another homeowner added; “We ask for a description of what can we expect from these CHO workers? They would never give us. So, there is no template for conflict resolution”.

#### Addressing power relations and philosophical differences

Homeowners were also concerned about power struggle issues and how to deal with the situation. During FGDs, homeowners disclosed that the ‘Profit’ versus ‘non-profit’ philosophy created a conflict of interest, leading to power struggle issues.

A homeowner suggested;

So, for a ‘not for profit’ to oversee a ‘for profit’ does not work, that’s important. You cannot do that. What’s happening with the agencies is that they are trying to run this like another not for profit program. We as individual business owners are losing our identity… they don’t understand that we are not for non-profit, that we own the beds that we purchased; we’re private business.

Another homeowner elaborated;

I’m saying that because they’re administering the program, [referring to Community Agencies] and they’re there to support the residents on one hand, but they’re also there now to ensure compliance of the contract which puts them in a power position. So, it should not be the same agency doing both of those

## Discussion

The current study explored the views of homeowners in relation to the CHO and changes related to policies and practices, to provide further insights for improving the program in Southwestern Ontario, Canada.

Thematic analysis of the homeowner data yielded five (5) major themes. These include general impressions about the CHO intervention, perceived social, economic, and health outcomes, enablers of the modernization program, challenges to the CHO implementation program, and suggestions for future implementation.

Supportive housing is generally reported to be effective among vulnerable populations faced with complex life challenges such as individuals with persistent mental illness or families experiencing homelessness [18, 33–35]. Housing challenges among persons with mental illness including addiction problems have been identified as a complex social problem of national importance [18], with human rights implications [34, 36, 37] that require urgent action. It is worth noting however that housing challenges among marginalized individuals continue to receive various societal and governmental policy attention. Despite these continued efforts by both provincial and federal governments, towards implementing supportive housing programs, homelessness, and housing instability continue to rise.

Homeowner participants of the current study expressed positive opinions about the modernization process of the HSC to CHO. The homeowners held positive perceptions of CHO. They also expressed a high level of optimism about the success of the housing program due to the cordial interpersonal relationships among tenants, staff, and homeowners. Such strong relationships among all stakeholders including landlords and the network of tenants (consumers), clinicians (staff), and managers are vital. Effective relationships in group homes have been found to encourage landlords to readily consult with the rest of the service team members when problems arise in the homes [10]. Again, Brackertz and colleagues, posit that developing healthy relationships with landlords helps to mitigate challenges that are associated with housing availability for tenants in the homes.

The ‘good feelings’ about how the CHO program was implemented transcended most homes due to the extra coverage (support) the homeowners were getting under the new housing intervention compared to the old program (the HSC). For instance, under the new housing intervention, homeowners get paid some money when a tenant moves in and when they incur an empty bed due to someone moving out of the house. This gesture by the stakeholders aligns with positive reinforcement theories of motivation as espoused by Thorndike [38] where homeowners would be more encouraged to accept new tenants into housing while supporting them throughout the recovery trajectory. Focus group data analysis of the homeowner perspectives revealed improved social, economic, and health outcomes for the tenants. According to the homeowners, such improvements were due to enhanced community involvement and socialization of their tenants, as well as the financial support they were receiving. The noticeable enhancement in quality of life and the associated empowerment that tenants were experiencing resulted from these enhanced activities and support [19].

Findings from the study established that even though the homeowners had positive impressions about the CHO program in relation to the benefits that accrued to them, some still encountered problems and provided suggestions to further improve the existing program. During the implementation of the CHO, the participants encountered problems that have implications for the quality of life and well-being of their tenants [2], as poor housing conditions due to limited resources were more likely to result in stress and burnout for both staff and homeowners [39] and leading to high turnover [40]. The homeowners also expressed concerns about the unhealthy behavior (lifestyle) of tenants, as well as the limited financial literacy of tenants and staffing problems. Poor housing conditions negatively affect the health [7–9] and well-being [2] of tenants. Therefore, the study findings underscore the need to engage with homeowners regularly to understand their peculiar difficulties for redress.

Supportive housing is a combination of housing and services intended as a cost-effective way to help people live more stable and productive lives [17]. Supportive housing services cannot thrive without the direct engagement of homeowners who are the primary custodians of the homes. The current findings have implications for future housing services, planning, and choice of homes for implementing the CHO program, especially in other jurisdictions in Ontario.

Overall, this CHO study expands on the Housing First literature in terms of (1) tenants receiving needs-driven supports, (2) tenants receiving improved financial support to cater for their needs (3) teamwork/collaboration between stakeholders i.e., homeowners, social workers, and the in-house support staff and (4) structured tenant community involvement and socialization activities for improved empowerment and integration. In all, the study underscores a need for good communication, enhanced resource allocation, and the involvement of homeowners during the planning and execution of all program activities in the participating homes for improved outcomes.

### Implications

Despite the challenges that homeowners experienced, study findings revealed that the participants perceived the CHO program as beneficial for the growth and development of their tenants toward social integration. The successful implementation of the CHO in the London-Middlesex area, emphasizes the need for future implementation of the CHO provincially.

Homeowners’ perspectives on the CHO program provide key insights for wide stakeholder involvement and consultation that includes homeowners’ views regarding their input for the successful implementation of the CHO in other jurisdictions. The study also provides tips for policymakers and Community Mental Health Agencies towards strategic planning along with needed investment that is more tailored to the needs assessment of each home for optimum services and outcomes. Overall, the CHO emphasizes housing as a key step towards recovery for persons with mental illness and addiction.

### Strengths and limitations

One of the strengths of this study is the fact that it highlights the active involvement of homeowners in all stages of the CHO implementation. Not only was the study undertaken in the participants’ settings, but it also provided the homeowner participants an opportunity to express their views (in terms of their experience) with the previous model (HSC) and the modernized program (CHO) respectively. The ethnographic approach facilitated homeowners’ participation that generated relevant findings that helped to uncover the actual nature of the program as well as enablers and barriers to the effective implementation of the modernized program.

However, we acknowledge that the findings of this study are based on the experiences of a group of homeowners in relation to the implementation of a particular housing program in response to a peculiar situation. Therefore, these findings may only transfer to contexts and settings like this study’s milieu. We also believe that personal experiences are time and context-dependent. Therefore, the homeowner’s views about the CHO program may change with time. As such, there is a need for continuous evaluation to ascertain other realities that may not have been captured by the current study for update or redress.

## Conclusion

Supportive housing models such as the CHO constitute an effective pathway to ending chronic homelessness for people with mental illness or addictions. Homeowners play significant roles in making houses to become comfortable and secure homes for tenants.

A more efficient and expanded CHO program will need the effective collaboration of all stakeholders including homeowners for successful empowerment and integration of persons with mental illness and addiction. It is hoped that standardizing such organizational functioning of the CHO will improve transparency and collaboration between stakeholders while addressing power relations and philosophical differences that homeowners perceive to exist.

## Data Availability

Datasets generated and/or analyzed during the current study are not publicly available due to participant privacy but are available from the corresponding author upon reasonable request.

## Abbreviations

CHO: Community Homes for Opportunity
HSC: Homes for Special Care
